# Medical History from the Earliest Times

**Published:** 1892-06-04

**Authors:** E. T. Withington


### Medical History from the Earliest Times.

By E. T. Withington, M.A., M.B. Oxon.

### XI.—THE SCHOOLS OF COS AND CNIDUS.

Among the relics of ancient medical writings preserved by Caelius Aurelianus is a series of arguments by which Euryphon, of Cnidus, tries to show that pleurisy is an affection of the substance of the lung, but which are refuted in detail by Soranus the Methodist. Euryphon was the most prominent member of the Asclepiad school of Cnidus, and lived at, or shortly before, the time of Hippocrates. His treatment seems to have been as unfortunate as his pathology, for a certain Cinesias is described as being "thin as a skeleton, his legs like reeds, his chest still full of pus, and his ribs covered with scars from the cautery irons of Euryphon."

The above indicates the leading characteristics of the physicians of Cnidus, whose grand aim and motto appear to have been "accurate diagnosis and vigorous treatment." But, unhappily, the want of modern methods of precision caused their diagnosis to result only in a long list of doubtful diseases. and the heavy artillery of their treatment being thus fired in the dark, may have produced results often more disastrous than successful.

The Asclepiadæ of Cos, under the beneficent influence of the great Hippocrates, followed different principles. They cared more for the general state of the individual patient than for the discovery and distinction of separate diseases, and they believed, though perhaps not to the extent sometimes asserted, in the *vis medicatrix naturæ*. We must not, however, make too much of these contrasts, and historians have, perhaps, somewhat exaggerated the antagonism between the two divisions of the great medical guild. The father of medicine has briefly pointed out the fundamental error of his Cnidian colleagues—they neglected "prognosis." What he meant by this term has already been discussed.

For the credit of Euryphon it should be added that he is said to have introduced percussion as a means of distinguishing tympanites from dropsy, and to have first prescribed milk, especially asses' milk, in cases of phthisis. Another distinguished Cnidian was Ctesias, for seventeen years physician to King Artaxerxes II., and historian of Persia, in which work he describes the tragic fate of his fellow Asclepiad, Apollonides of Cos, who, having given immoral medical advice to the Princess Amytis, was tortured for two months, and finally buried alive.

The Hippocratic writings stand in isolated grandeur at the entrance to the second great division of our subject like one of those triumphal arches of Titus or Constantine, which rise amid the ruins of ancient Rome, for the works of later physicians have almost entirely disappeared, leaving a barren interval of nearly four centuries, till we come to the days of Celsus, Pliny, and Dioscorides. Besides the works of Hippocrates, the collection comprises contributions from his pupils and other members of the school of Cos, together with a few which may be attributed to the Asclepiadæ of Cnidus; but the following very brief outline of the medical doctrines therein contained is taken mainly from those treatises which may reasonably be ascribed to the father of medicine himself.

Hippocrates knows the four humours—blood, phlegm, black and yellow bile, and their corresponding four qualities—heat, cold, dryness, and moisture; and he admits with Alcmæon and the other Greeks that diseases may sometimes be due to a predominance of one of the former. But when told that this acts by producing an excess of some particular quality, such as heat, and is to be cured by drugs which have an excess of the opposite quality, *e.g.*, cold, he demurs. The doctrine is too vague for him, he does not know which drugs are "cold" and which "hot," and thinks that such theories are best left to sophists. In their place he proposes a hypothesis of great ingenuity. May not the humours, when imperfectly or disproportionately mixed, act as irritants to the body, just as uncooked food acts as an irritant to a healthy stomach, and just as we get rid of the evils of uncooked food by cooking it, may not diseases be cured, and the humours reduced to their normal state of mixture—"crasis"—by a sort of internal coction—"pepsis"? The agent of this coction may, he thinks, be the innate heat of the body, the "thermon emphyton" of, Heraclitus, but he prefers to call it "Nature." Nature then, tends to restore the normal state, and this is often accomplished on a particular day of the disease, and accompanied by copious sweats or other excretions. Here is the famous "*Vis medicatrix naturæ*"; but shall the physician stand idle, with his hands wrapped in his chiton, while this is going on? No, by Æsculapius; let him carefully observe the aspect of the patient and his position in bed; let him use his hands to feel the temperature of his body, and his eyes to observe the character of his excretions; let him even put his ear to his chest, and he may distinguish the leather-like rub of pleurisy, or by shaking him obtain the splash of pneumo-hydrothorax. Strangely enough, the pulse, so clearly described by the ancient Nebsecht, and so fully worked out by the later Herophilus, is hardly mentioned by Hippocrates. But having thus examined his patient, how shall the physician treat him? Let him look first to his general surroundings and see that they are at least not unfavourable, and especially attend to his diet, remembering that as uncooked food is to a healthy man, so is ordinary food to one with acute disease. Let him also use drugs, bleeding, &c., if he thinks he may thereby assist nature in her efforts; by so doing he shall take his proper position as her servant, for "our natures are the physicians of diseases."

Hippocrates, however, is not a mere humouralist, even in this modified form, for he holds that diseases may arise also from alterations in the structures of the body, and especially from external influences, such as climate, seasons, and the like, which he sums up under the term "constitution," an idea afterwards much developed in the writings of our English Hippocrates, Thomas Sydenham; nor is he anxious to defend the above or any other hypothesis, and thinks that those who spend time in so doing only show their own volubility.

We cannot stay longer even with the greatest of physicians, and must entirely pass over the surgery of Hippocrates, which, considering the practical absence of anatomical knowledge, is yet more admirable than his medicine. Still, even the above imperfect outline of a part of his work may suffice to show that the almost enthusiastic reverence which so many great physicians have paid to Hippocrates, was not altogether unjustified.

And his fame was not confined to physicians, nor even to Europe. The mediæval romancers classed Ypocras with Aristotle and Virgil as a great sorcerer, and delighted to tell how, in spite of his wisdom, he was beguiled by fair women into doubtful situations, and finally poisoned by his jealous wife. The Arabs adopted him as their own under the name Bucrat—Father of Crat—and an early Oriental traveller was told by an Arab doctor that no Christian nation could boast of such a physician as was Bucrat, who, he declared, was the greatest of the hakims, and lived shortly before Avicenna. A learned German even believes that the great Hindoo, Susruta, is none other than Hippocrates, whose name has been confounded with that of Socrates, and corrupted thus—Bucrata-Sucrata-Susruta.

We still have far to travel through many ages and countries, but in all our future path the doctrine of the great Asclepiad will shine above us like a guiding star, dimmed indeed sometimes by the mists of theories and systems, covered at other times by the clouds of ignorance and superstition, yet still shining, and still sought for by all who have ever attempted or achieved progress in medicine.

